# Dextrins from Maize Starch as Substances Activating the Growth of *Bacteroidetes* and *Actinobacteria* Simultaneously Inhibiting the Growth of *Firmicutes*, Responsible for the Occurrence of Obesity

**DOI:** 10.1007/s11130-016-0542-9

**Published:** 2016-05-07

**Authors:** Renata Barczynska, Janusz Kapusniak, Mieczyslaw Litwin, Katarzyna Slizewska, Mieczyslaw Szalecki

**Affiliations:** Institute of Chemistry, Environmental Protection and Biotechnology, Jan Dlugosz University in Czestochowa, Armii Krajowej 13/15, 42-200 Czestochowa, Poland; The Children’s Memorial Health Institute, Warsaw, Poland; Institute of Fermentation Technology and Microbiology, Faculty of Biotechnology and Food Sciences, Technical University of Lodz, Lodz, Poland; Faculty of Health Sciences, UJK, Kielce, Poland

**Keywords:** Dextrin, Prebiotics, Microbiota, *Firmicutes*, *Bacteroidetes*

## Abstract

Unarguably, diet has a significant impact on human intestinal microbiota. The role of prebiotics as substances supporting the maintenance of appropriate body weight and reducing the demand for energy *via* stimulation of the growth of beneficial microbiota of the gut and formation products such as short-chain fatty acids, is more and more often highlighted. The objective of this study was to evaluate whether dextrins from maize starch resistant to enzymatic digestion stimulate the growth of *Bacteroidetes* and *Actinobacteria* strains representing a majority of the population of colon microbiota in lean individuals and limit the growth of *Firmicutes* bacterial strains representing a majority of the population of colon microbiota in obese individuals. The study was conducted with the use of *in vitro* method, using isolates from faeces of children characterized by normal weight, overweight and obesity. It was demonstrated that dextrins from maize starch equally efficient stimulate the growth of the isolates derived from normal-weight, overweight and obese children, and therefore may be added to foods as a beneficial component stimulating growth of strains belonging to *Actinobacteria* and *Bacteroidetes* for both overweight, obese and normal-weight children.

**Electronic supplementary material:**

The online version of this article (doi:10.1007/s11130-016-0542-9) contains supplementary material, which is available to authorized users.

## Introduction

According to Roberfroid *et al.* 2010 [1] prebiotics are defined as “the selective stimulation of growth and/or activity(ies) of one or a limited number of microbial genus(era)/species in the gut microbiota that confer(s) health benefits to the host”. Efficacy of prebiotics is owed to the fact that they are not hydrolyzed and absorbed in the upper sections of the gastrointestinal tract, so they reach the colon in uncharged form where they constitute substrates for beneficial bacteria [2]. More and more often, the role of prebiotics as substances supporting maintenance of appropriate body weight and reducing the demand for energy, through stimulating the growth of beneficial intestinal microbiota and the formation of such products as short-chain fatty acids (SCFA), is highlighted. Many studies indicate that obesity has a multifactorial background (genetic, environmental, social), and one of such factors are changes in the composition and metabolic function of the intestinal microbiota. It is acknowledged that it is particularly important to maintain the right proportion of *Bacteroidetes* and *Firmicutes* strains [3–7].

Studies conducted by the group of Ley, Backhed and De Filippo also indicate that obesity in humans is associated with the composition of intestinal microbiota [3, 8, 9]. Backhed *et al.* [8, 10] determined the contribution of *Bacteroidetes* and *Firmicutes* in obese and normal-weight mice and found that the proportion of *Bacteroidetes* is significantly lower in obese mice (20 %), while in normal-weight mice, the contribution of these bacteria was higher and accounted for up to 40 %. In turn, Fleissner *et al.* [11] demonstrated that diet rich in animal fats and low content of fiber administered to mice results in a reduction of the quantity of *Bacteroidetes* strains and the growth of *Firmicutes*. Moreover, studies aimed at explanation whether the use of prebiotics may promote a feeling of satiety and reduction of hunger were conducted [12, 13]. It was demonstrated that one can associate the positive effect of prebiotics with modulation of intestinal microbiota, particularly with the production of SCFA and increased level of PYY (neuropeptide anorexigenic, this peptide is synthesized and secreted by the L-cells of the ileum and colon exhibiting stimulating effect on satiety center) and GLP-1 (glucagon-like peptide-1) resulting in reduced glycemia, insulin resistance and body fat, increasing the feeling of satiety [14–16].

The objective of the study was to evaluate whether resistant dextrins from maize starch obtained with the use of citric or tartaric acids are capable of stimulating the growth of *Bacteroidetes* and *Actinobacteria* strains representing the majority of the colon microbiota in lean individuals and at the same time reducing the growth of *Firmicutes* bacteria representing a majority of the colon microbiota in obese individuals.

## Materials and Methods

### Bacteria

In the study, *Lactobacillus, Bifidobacterium, Prevotella, Clostridium, Bacteroides* intestinal bacteria were isolated from faeces of 20 normal-weight children and 20 overweight and obese children—patients from the Institute “Monument—Children’s Health Center” in Warsaw. Selection of the study group was based on the criteria of the International Obesity Task Force (IOTF) developed by Cole *et al.* [17].

### Starch Formulations

Dextrin was prepared at the Institute of Chemistry, Environmental Protection and Biotechnology, Jan Dlugosz University in Częstochowa. Dextrins were obtained from maize starch subjected to piroconversion and chemical modification: citric dextrin (K1) produced as a result of maize starch heating at 130 °C for 180 min with the addition of hydrochloric acid as a catalyst and citric acid as crosslinking agent; tartaric dextrin (K2) obtained as a result of maize starch heating at 130 °C for 180 min with the addition of hydrochloric acid as a catalyst and tartaric acid as crosslinking agent.

### Determination of Quantity of Bacteria

Cultivation of the joint cultures of *Lactobacillus, Bifidobacterium, Bacteroides, Clostridium, Prevotella* intestinal bacteria isolated from faeces of 20 normal-weight children and 20 overweight and obese children, was carried out in media containing dextrins from maize starch. Prepared a suspension of bacteria that the number of cells of particular bacteria was established at about 107–108 cfu/ml, the number of cells of particular bacteria was established at about 10^7^–10^8^ cfu/m (colony forming units/m), which corresponds to the number of cells of these microorganisms in the initial section of the colon. Biomass bacteria was transferred into 100 ml medium [18] containing K1 or K2 dextrin as the sole carbon source. The cultures were incubated at 37 °C for 168 h. Immediately after inoculation, and after 24 h of incubation, cultivation according to Koch’s plate method was carried out from each bacterial culture using the appropriate selective media. *Clostridium* strains were cultured with the use of differential reinforced clostridial broth (DRCM, MERCK, Darmstadt, Germany), *Bacteroides* strains with the use of Schaedler medium with gentamycin antibiotic (BioMerieux, Marcy I’Etoile, France), *Prevotella* strains using Brucella medium composed of antibiotics such as Polymyxin B, Bacitracin, Cycloheximide, Nalidixic acid, Nystatin, Vancomycin (BioMaxima, Poland), reinforced clostridial agar (RCA) [19] with the addition of dicloxacillin antibiotic was used for *Bifidobacterium*, *Lactobacillus* strains were cultured using Rogosa medium (MERCK, Darmstadt, Germany).

### Determination of Lactic Acid, SCFA and BCFA Using HPLC

Lactic acid, short-chain fatty acids (acetic, propionic, butyric, formic, and valeric) and branched fatty acids (BCFA; isovaleric and isobutyric) were determined by high-performance liquid chromatography (HPLC) using a Surveyor chromatograph (Thermo Scientific) and an Aminex HPX-87H column (300 × 7.8 mm) from Bio-Rad Aminex® with sulfonated divinyl benzene-styrene copolymer support. The following analytical parameters were used: 300 × 7.8 mm Aminex HPX-87H column, mobile phase 0.005 M H_2_SO_4_, 210 nm UV detector, injector valve with a sample loop, injection volume 10 μl, column temperature 60 °C, flow rate 0.6 μl/min, analysis of a single sample 35 min. Samples with known concentrations of the acids (0; 0.125; 0.25; 0.50; 0.75 and 1 % acid/ml) were analyzed with HPLC in order to obtain calibration curves showing acid concentration to peak area ratios.

### Statistical Analysis

The results were evaluated with W-Shapiro Wilk test assessing normality of the distribution of the results. Due to the deviation from the normal distribution, further analysis was based on U Mann–Whitney test. Statistical significance was established at *p* < 0.05. The statistical analysis was performed using STATISTICA 10.0 software (StatSoft, Inc.).

## Results and Discussion

**Fig. 1:**
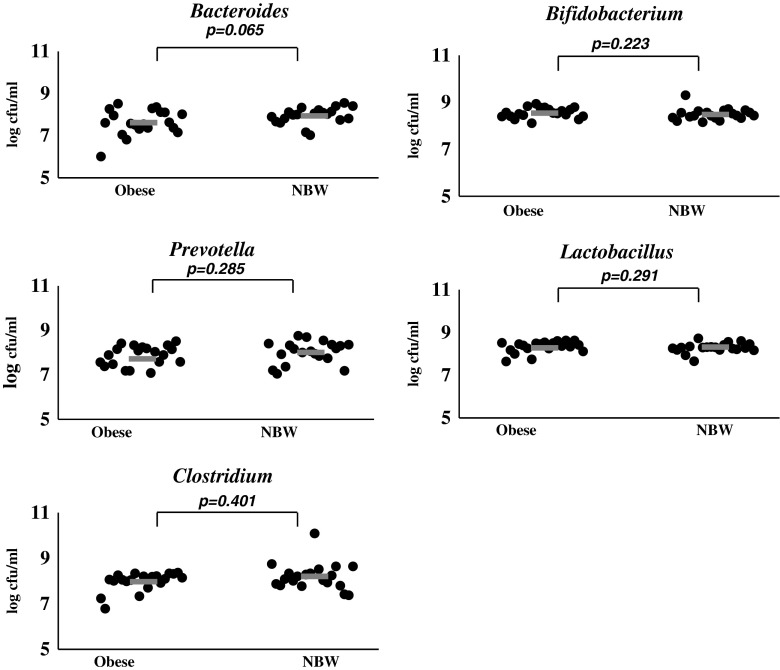
The number of tested strains isolated from faeces of overweight, obese and normal-weight children in medium supplemented with K1 dextrin. *NBW* normal body weight, − Average

The highest efficiency of stimulation of *Bifidobacterium* and *Lactobacillus* strains isolated from both, faeces of overweight and obese children (8.54 and 8.32 log cfu/ml, respectively) as well as from faeces of normal-weight children (8.48 and 8.28 log cfu/ml, respectively), was observed for dextrin obtained from maize starch with the use of citric acid (K1). The number of *Prevotella* and *Clostridium* strains isolated from faeces of overweight and obese children was comparable and accounted for 7.85 and 7.97 log cfu/ml respectively, while for normal-weight children it was estimated at 8.01 and 8.20 log cfu/ml, respectively. *Bacteroides* strains isolated from faeces of children with overweight, obesity and normal weight used K1 dextrin with the lowest efficacy. However, the quantity of strains isolated from faeces of normal-weight children was slightly higher and was 7.94 log cfu/ml in comparison to the culture of strains isolated from faeces of overweight and obese children (7.62 log cfu/ml) (Fig. 1).

**Fig. 2:**
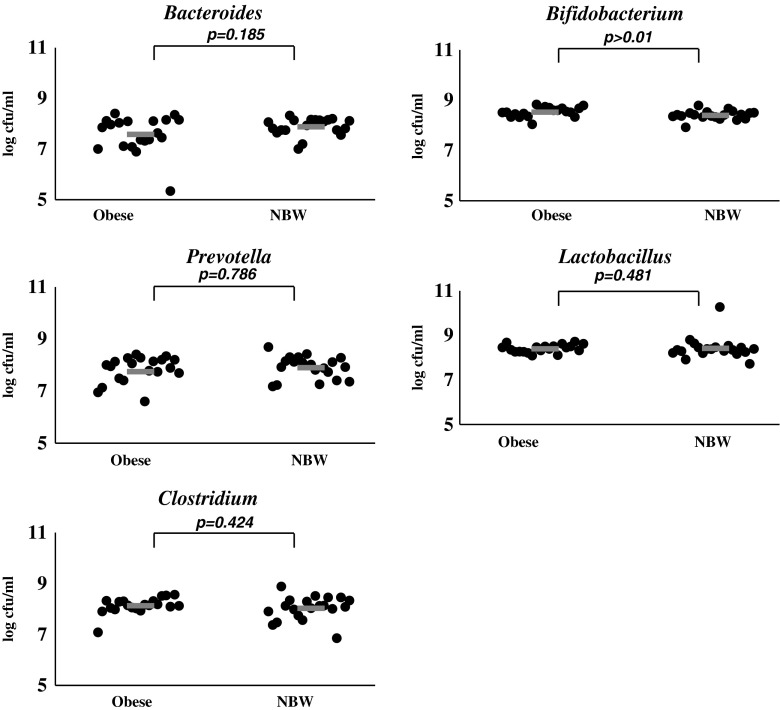
The number of tested strains isolated from faeces of overweight, obese and normal-weight children in medium supplemented with K2 dextrin. *NBW* normal body weight, − Average

Dextrin obtained from maize starch with the use of tartaric acid (K2) best stimulated the growth of *Bifidobacterium* strains isolated from faeces of overweight and obese children, the quantity of these strains after 24th hour of incubation was 8.53 log cfu/ml. *Bifidobacterium* strains isolated from faeces of normal-weight children and *Lactobacillus* strains isolated from faeces of normal-weight, overweight and obese children equally well used K2 dextrin as a carbon source, their number was very similar and accounted for 8.4–8.42 log cfu/ml. The number of *Prevotella* strains isolated from both groups of children in the culture supplemented with K2 dextrin was similar and accounted for 7.83 and 7.90 log cfu/ml, similarly as *Clostridium* strains after 24th hour incubation with K2 dextrin, as their quantity was estimated at 8.03 and 8.13 log cfu/ml. In analogy to the culture supplemented with K1 dextrin, *Bacteroides* strains isolated from faeces of overweight, obese or normal-weight children used K2 dextrin with the least efficiency, the quantity of these strains after 24th hour of incubation was 7.58 and 7.88 log cfu/ml, respectively (Fig. 2).

## The Percentage Contribution of Bacteria Belonging to *Firmicutes*, *Bacteroidetes* and *Actinobacteria* Types

The percentage composition of the tested types of *Firmicutes*, *Bacteroidetes* and *Actinobacteria* bacteria was very similar in media supplemented with K1 and K2 dextrins independently of the dextrin used and independently of whether the strains were isolated from faeces of normal-weight, overweight or obese children. Bacteria belonging to *Firmicutes* type accounted for 40–41 % of the population of bacteria in culture, bacteria belonging to *Bacteroidetes* type accounted for 38–39 % of the population, while bacteria belonging to the type *Acinobacteria* type accounted for 21–22 % of all strains tested (Fig 3. online resource).

## SCFA and BCFA in Faeces of Obese and Normal-Weight Children

After 24 h of joint cultivation of bacteria isolated from faeces of obese children in the medium supplemented with dextrin (K1), lactic acid concentrations ranged from 1.61 to 6.88 mg/g of faeces (3.55 mg/g of faeces on average). SCFA concentrations ranged from 3.63 to 8.48 mg/g of faeces (5.60 mg/g of faeces on average) whereas BCFA concentration from 0.18 to 0.52 mg/g of faeces (0.11 mg/g of faeces on average). The lowest quantity of valeric acid (0.42 mg/g of faeces), butyric acid (0.52 mg/g of faeces) and formic acid (0.88 mg/g of faeces) was found in culture of strains isolated from faeces of obese children containing K1 dextrin. The average amount of acetic and propionic acid was established at 2.65 and 1.13 mg/g of faeces. Among BCFA acids, isobutyric acid whose concentration was 0.11 mg/g of faeces was predominant, whereas the concentration of isovaleric acid was lesser and accounted for 0.04 mg/g of faeces on average. In the culture of the same strains but with the addition of dextrin (K2), lactic acid concentrations ranged from 2.72 to 4.96 mg/g of faeces (average concentration of acid was 3.75 mg/g of faeces, similar to the amount of lactic acid in the culture with K1 dextrin). SCFA concentrations ranged from 3.88 to 8.67 mg/g of faeces (5.88 mg/g of faeces on average), while BCFA concentration from 0.18 to 0.28 mg/g of faeces (0.23 mg/g of faeces on average). The lowest amount of butyric (0.39 mg/g of faeces) and valerianic acid (0.40 mg/g of faeces) was found in strains isolated from faeces of obese children in which supplementation with K2 dextrin was used. The average amount of formic and propionic acid was comparable (1.01 and 1.20 mg/g of faeces). Among SCFA, the highest amount of acetic acid (2.86 mg/g of faeces) was found in strains isolated from faeces of obese children in which supplementation with K2 dextrin was used. Among BCFA, isobutyric acid whose concentration was 0.23 mg/g of faeces was predominant, whereas the concentration of isovaleric acid was lower and accounted for 0.02 mg/g of faeces on average. In the joint culture of bacteria isolated from faeces of obese children in the medium supplemented with K2 dextrin, average amount of lactic acid and SCFA was higher by about 5 % in comparison to mean values for these acids in culture supplemented with K1 dextrin, while the average amount of BCFA was higher by about 50 %. After 24 h of joint culture of bacteria isolated from faeces of children with normal body weight in the medium supplemented with dextrin (K1), lactic acid concentration ranged from 3.06 to 9.11 mg/g of faeces (6.04 mg/g of faeces on average). SCFA concentration ranged from 4.67 to 17.00 mg/g of faeces (9.79 mg/g of faeces on average), while BCFA concentration from 0.21 to 1.50 mg/g of faeces (0.42 mg/g of faeces on average). The lowest amount of butyric acid (0.80 mg/g of faeces) was found in strains isolated from faeces of children with normal body weight in which supplementation with K1 dextrin was used. The average amount of formic and pentanoic acid was similar and accounted for 1.30 and 1.46 mg/g of faeces. Among SCFA acetic (4.19 mg/g of faeces on average) and propionic acid (2.01 mg/g of faeces on average) were the highest in strains of culture isolated from faeces of normal-weight children in which supplementation with K1 dextrin was used. Among BCFA, isobutyric acid whose concentration was 0.41 mg/g of faeces was predominant, while the concentration of isovaleric acid was much lower and accounted for 0.04 mg/g of faeces on average. In the culture of the same strain however supplemented with K2 dextrin, lactic acid concentration, SCFA and BCFA was similar as in culture with K1 dextrin. The amount of SCFA ranged from 5.59 to 15.80 mg/g of faeces (9.40 mg/g of faeces on average), whereas BCFA concentration from 0.21 to 1.32 mg/g of faeces (0.50 mg/g of faeces on average). Lactic acid was predominant and its average amount was 5.67 mg/g of faeces. The lowest amount of butyric (0.82 mg/g of faeces) and valeric (0.91 mg/g of faeces) acids was reported, while the highest concentration was found for acetic acid (4.27 mg/g of faeces on average). The average amount of formic and propionic acids was estimated at 1.10 and 1.99 mg/g of faeces, respectively. Among BCFA, isobutyric acid whose concentration was 0.45 mg/g of faeces was predominant, whereas the concentration of isovaleric acid was much lower (0.02 mg/g of faeces on average). In the joint culture of bacteria isolated from faeces of children with normal body weight in the medium supplemented with K1 dextrin, average amount of lactic acid was decreased by about 6 % in comparison to the average amount of these acids in culture with K2 dextrin, while the amount of SCFA was decreased by 4 %. The average amount of BCFA in medium with the addition of K1 dextrin was decreased by about 16 % compared to the culture with K2 dextrin. It was observed that the average amount of lactic acid in the culture with isolates from children with normal body weight was higher by 41 % (medium supplemented with K1 dextrin) and 34 % (medium supplemented with K2 dextrin) compared to the concentration of this acid in the culture of isolates from children with overweight and obesity. Similar relationship was observed for SCFA, their concentration was higher by 43 % (K1) and 39 % (K2) in cultures of isolates from children with normal body weight in comparison to the isolates from obese children, also BCFA concentration was higher by 74 % (K1) and 54 % (K2) (Tab 1. online resource).

In the studies, one used dextrins derived from maize starch, in which one demonstrated the occurrence of approximately 70 % of resistant fraction. Enhanced resistance to enzymatic digestion was caused by the presence of bonds other than typical for starch, particularly α-(1 → 4)-glycosidic bonds [20]. The product obtained with newly formed bonds was not metabolized in the upper section of the digestive tract, but was transferred into the colon, where constituted the source of carbon and energy for the intestinal bacteria. This fact has been investigated and dextrins were used as a carbon source in the culture medium of bacteria isolated from faeces of normal-weight, overweight and obese children. It was shown that all of the tested bacterial strains isolated from obese, overweight and lean children used resistant dextrins as a carbon source, however in different degrees. Resistant dextrins from maize starch were used by *Lactobacillus* and *Bifidobacterium* strains with the highest efficiency, while the lowest efficiency was reported for *Clostridium* and *Bacteroides*, which is consistent with previously published studies [21, 22].

De Filippo *et al.* [9] were demonstrated that, irrespective of the diet used, *Actinobacteria*, *Bacteroidetes* and *Firmicutes* types of bacteria were dominant in the digestive tract, however their proportions were different and dependent on the diet. In children from Burkina Faso, *Actinobacteria* and *Bacteroidetes* were predominant and accounted for 10.1 and 73 % respectively, while *Firmicutes* bacteria accounted for 10 %. In children from Florence, one found higher body weight and higher incidence of obesity and intestinal microbial system was different in comparison to children from agricultural lands in Africa. It has been shown that *Firmicutes* bacteria were predominant (51 %), while *Actinobacteria* and *Bacteroidetes*, accounted for 6.7 and 27 %, respectively. In our studies, we demonstrated that the use of dextrins from maize starch obtained in the presence of citric acid (K1) and tartaric acid (K2) during joint cultivation of *Bifidobacterium, Lactobacillus, Prevotella, Clostridium, Bacteroides* strains stimulates the growth of strains belonging *Actinobacteria* and *Bacteroidetes* types (59–60 %), while stimulation to a lesser extent was observed for *Firmicutes* (40–41 %). Regardless whether the strains were isolated from faeces of normal-weight, overweight or obese children.

In human organism, SCFA are produced as a result of anaerobic decomposition of dietary fiber and starch, so they are products of fermentation conducted by anaerobic bacteria colonizing cecum and colon. Due to the important role of SCFA in the human organism and the possible impact on the development of obesity, it is important that the addition of dextrins does not change the normal SCFA profile. SCFA exhibit many beneficial functions. Butyric acid stimulates the growth of intestinal epithelial tissue, nourishes intestinal cells, affects their proper maturation and differentiation. Propionic acid has a positive effect on the growth of hepatocytes, whereas acetic acid on the development of peripheral tissues. SCFA regulate glucose and lipid metabolism, stimulate proliferation and differentiation of intestinal enterocytes, contribute to a decrease in pH of the intestinal contents and thereby promote the absorption of minerals by increasing their solubility [23, 24].

Resistant dextrins from maize starch were metabolized *via* lactic, butyric, propionic and mixed fermentation. Dextrin from maize starch (K1 and K2) similarly stimulated the growth of bacteria of *Actinobacteria, Bacteroidetes* and *Firmicutes* types, while the concentration of metabolites was dependent on the type of bacteria, their origins and the dextrin used. More SCFA have been detected in culture of isolates derived from children with normal body weight in comparison to children with obesity and overweight. It should, however, be noted that obese children excrete far fewer compounds that may constitute a source of energy compared to individuals with normal weight [25]. Despite non-digestible carbohydrates, fermentation by colonic microbiota may also be attributed to proteins and amino acids, resulting in the formation of SCFA or BCFA, to which isobutyric isovaleric acids belong to. Phenols, indoles and ammonia are also products which are toxic for human organism, indicating this type of fermentation as harmful [26, 27]. In my research as a consequence of fermentation of dextrins from maize starch, one reported very low concentration of BCFA, from 0.20 to 0.71 mg/g of faeces on average higher in cultures of isolates from children with normal body weight and lower in cultures of isolates derived from children with overweight and obesity.

## Conclusions

Dextrins obtained by heating maize starch at 130 °C for 180 min with the addition of hydrochloric acid as a catalyst and citric (K1) or tartaric acid (K2) as crosslinking agents equally well stimulate the growth of the isolates derived from normal-weight, overweight and obese children and resistant dextrin stimulated an increase in the concentration of SCFA and thus may be added to foods as beneficial (health-promoting) component stimulating growth of strains belonging to *Actinobacteria* and *Bacteroidetes* types for children with overweight and obesity and for normal weight children as well. Randomised controlled clinical study is needed to confirm dextrins efficacy as a health promoting components.

## Electronic supplementary material

Below is the link to the electronic supplementary material.

Fig 3 (DOCX 26 kb)

Tab 1 (DOCX 15 kb)

## Abbreviations

BCFA: Branched-chain fatty acids
DRCM: Differential reinforced clostridial broth
GLP-1: Glucagon-like peptide-1
HPLC: High-performance liquid chromatography
IOTF: International Obesity Task Force
K1: K2, dextrins (dietary fiber) preparations made from maize starch (citric acid in the K1 fiber preparation and tartaric acid in the K2 fiber preparation)
NBW: Normal body weight
PYY: Neuropeptide anorexigenic
RCA: Reinforced clostridial agar
SCFA: Short-chain fatty acids
