# Supplementary material for: Dextrins from Maize Starch as Substances Activating the Growth of Bacteroidetes and Actinobacteria Simultaneously Inhibiting the Growth of Firmicutes, Responsible for the Occurrence of Obesity
Source: Plant Foods Hum Nutr. 2016 May 7;71:190–6. doi: 10.1007/s11130-016-0542-9 (PMC4891389; doi:10.1007/s11130-016-0542-9)
Supplement: Supplementary file 1 — (DOCX 26 kb) [file 11130_2016_542_MOESM1_ESM.docx]

**b**

**a**

**d**

**c**

**Fig 3.** Percentage distribution of bacteria in culture with K1 dextrin, strains isolated (a) from overweight or obese children and (b) lean individuals; with K2 dextrin, strains isolated (c) from overweight and obese children or (d) lean individuals.
