# Supplementary material for: Dextrins from Maize Starch as Substances Activating the Growth of Bacteroidetes and Actinobacteria Simultaneously Inhibiting the Growth of Firmicutes, Responsible for the Occurrence of Obesity
Source: Plant Foods Hum Nutr. 2016 May 7;71:190–6. doi: 10.1007/s11130-016-0542-9 (PMC4891389; doi:10.1007/s11130-016-0542-9)
Supplement: Supplementary file 2 — (DOCX 15 kb) [file 11130_2016_542_MOESM2_ESM.docx]

**Tab 1.** SCFA and BCFA concentration in the culture with K1 dextrin, strains isolated from overweight or obese children and lean individuals; with K2 dextrin, strains isolated from overweight and obese children or lean individuals.

|  | Citric dextrin K1 | | | | | Tartaric dextrin K2 | | | | |
| --- | --- | --- | --- | --- | --- | --- | --- | --- | --- | --- |
|  | Obese children | | Slim children | | *p* | Obese children | | Slim children | | *p* |
|  | Acid concentration  [mg/g faeces] | Average  [mg/g faeces] | Acid concentration  [mg/g faeces] | Average  [mg/g faeces] |  | Acid concentration  [mg/g faeces] | Average  [mg/g faeces] | Acid concentration  [mg/g faeces] | Average  [mg/g faeces] |  |
| Lactic acid | 1.61-6.88 | 3.55 | 3.06-9.11 | 6.04 | >0.001 | 2.72-4.96 | 3.75 | 2.86-8.21 | 5.67 | >0.001 |
| SCFA |  |  |  |  |  |  |  |  |  |  |
| Acetic | 1.94-3.93 | 2.62 | 2.76-7.49 | 4.19 | >0.001 | 1.88-4.17 | 2.86 | 3.22-7.50 | 4.27 | >0.001 |
| Propionic | 0.81-1.52 | 1.13 | 0.75-2.67 | 2.01 | >0.001 | 0.87-1.62 | 1.20 | 0.77-2.52 | 1.99 | >0.001 |
| Butyric | 0.26-1.02 | 0.52 | 0.33-1.87 | 0.80 | >0.001 | 0.20-0.61 | 0.39 | 0.53-1.72 | 0.82 | >0.001 |
| Formic | 0.36-1.38 | 0.88 | 0.43-2.32 | 1.30 | >0.001 | 0.60-1.76 | 1.01 | 0.59-2.21 | 1.40 | >0.001 |
| Valeric | 0.25-0.61 | 0.42 | 0.37-2.72 | 1.46 | >0.001 | 0.31-0.48 | 0.40 | 0.46-1.85 | 0.91 | >0.001 |
| Total SCFA | 3.63-8.48 | 5.60 | 4.67-17.0 | 9.79 | >0.001 | 3.88-8.67 | 5.88 | 5.59-15.8 | 9.40 | >0.001 |
| BCFA |  |  |  |  |  |  |  |  |  |  |
| Isovalerian | 0.01-0.09 | 0.04 | 0.01-0.16 | 0.04 | NS | 0.01-0.04 | 0.02 | 0.01-0.18 | 0.05 | >0.001 |
| Isobutanoic | 0.17-0.42 | 0.11 | 0.20-1.34 | 0.41 | >0.001 | 0.17-0.24 | 0.20 | 0.20-1.14 | 0.45 | >0.001 |
| Total BCFA | 0.18-0.52 | 0.11 | 0.21-1.50 | 0.42 | >0.001 | 0.18-0.28 | 0.23 | 0.21-1.32 | 0.50 | >0.001 |
| SCFA:BCFA | 95:5-91:9 | 98:2 | 96:4-92:8 | 96:4 | - | 95:5-97:3 | 96:4 | 96:4-92:8 | 95:5 | - |
